# Supplementary material for: Recruitment of the cellular lipid transport protein CERT to C. psittaci inclusions regulates the timing of bacterial egress
Source: Sci Rep. 2025 May 25;15:18241. doi: 10.1038/s41598-025-02077-w (PMC12104389; doi:10.1038/s41598-025-02077-w)

Supplementary Information file

for Submission ID 49fd8d0c-1016-4a33-be85-aa45fdea0d37 (*Recruitment of the cellular lipid transport protein CERT to C. psittaci inclusions regulates the timing of bacterial egress*)

Author list:

Jana Scholz
scholzj@rki.de
Robert Koch Institute DE

Gudrun Holland
hollandg@rki.de
Robert Koch Institute DE

Michael Laue
lauem@rki.de
Robert Koch Institute DE

Sebastian Banhart
banharts@rki.de
Robert Koch Institute DE

Corresponding author:
Dagmar Heuer
heuerd@rki.de
Robert Koch Institute DE

Supplemental Figure S1: CCS formation under CERT-KO conditions is induced at 20 and 24 h pi and reduced at 72 h pi. (A) CCS formation in HeLa CERT-KO and KO control cells normalized to the inclusion number at 16 h pi. Cells were infected with *C. psittaci* (MOI 2), and at 16 h pi, sample was fixed for inclusion quantification based on Hsp60 staining. Medium was replaced at 16 and 20 h pi and CCS in the supernatant were visually quantified at 20 and 24 h pi, respectively. Data were normalized to the inclusion number at 16 h pi and shown as mean ± SEM; n = 2. **(B)** CCS formation in HeLa CERT-KO and KO control cells at 72 h pi. Cells were infected with *C. psittaci* (MOI 2), medium was replaced at 68 h pi and CCS in the supernatant were visually quantified at 72 h pi. Data are shown as mean ± SEM; n = 2.

Supplemental Figure S2: In contrast to *C. trachomatis* L2, *C. psittaci* does not recruit STIM1 to the chlamydial inclusion. (A) Representative immunofluorescence images of *C. psittaci*-infected HeLa cells (MOI 2) at 24 and 48 h pi. PFA-fixed cells were stained for *C. psittaci* and endogenous STIM1 using a mouse-anti-Hsp60 (Cy3) and a rabbit-anti-STIM1 (AF488) antibody, respectively. DNA was counterstained using DAPI. n = 3. (B) Representative immunofluorescence images of *C. trachomatis* L2-infected HeLa cells (MOI 2) at 24 h pi. PFA-fixed cells were stained for *C. trachomatis* L2 and endogenous STIM1 using a mouse-anti-Hsp60 (Cy3) and a rabbit-anti-STIM1 (AF488) antibody, respectively. DNA was counterstained using DAPI. n = 3.

Supplemental Figure 1

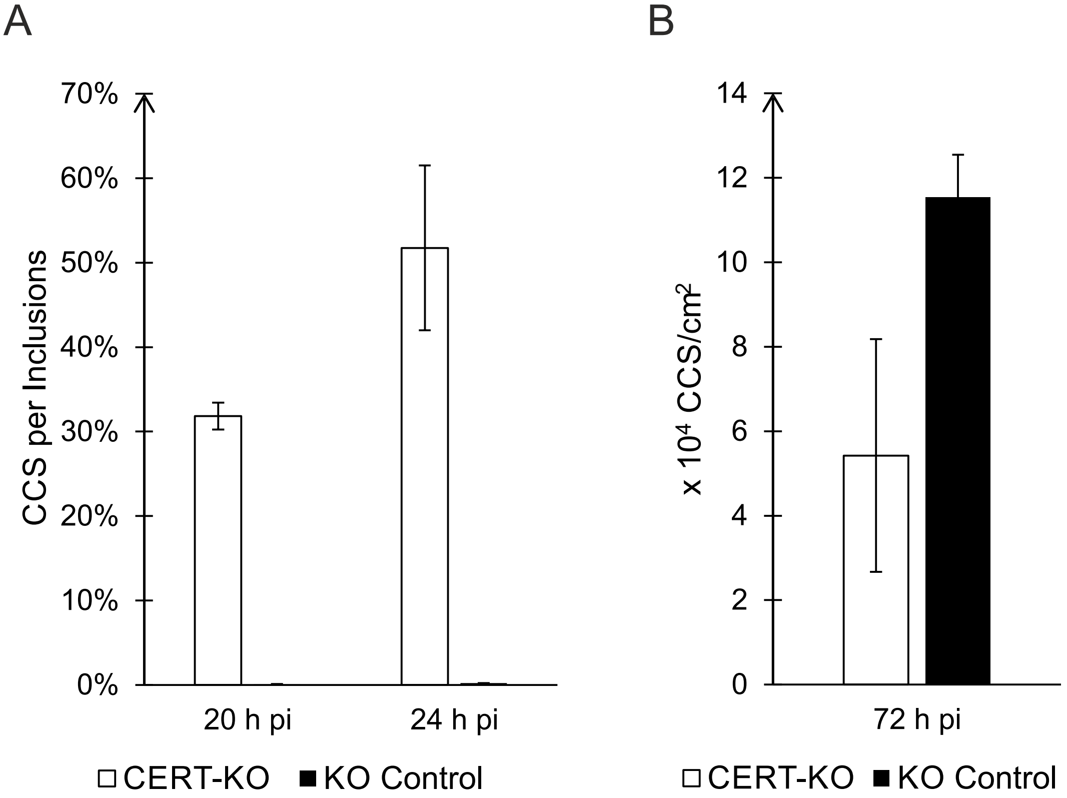

Supplemental Figure 2

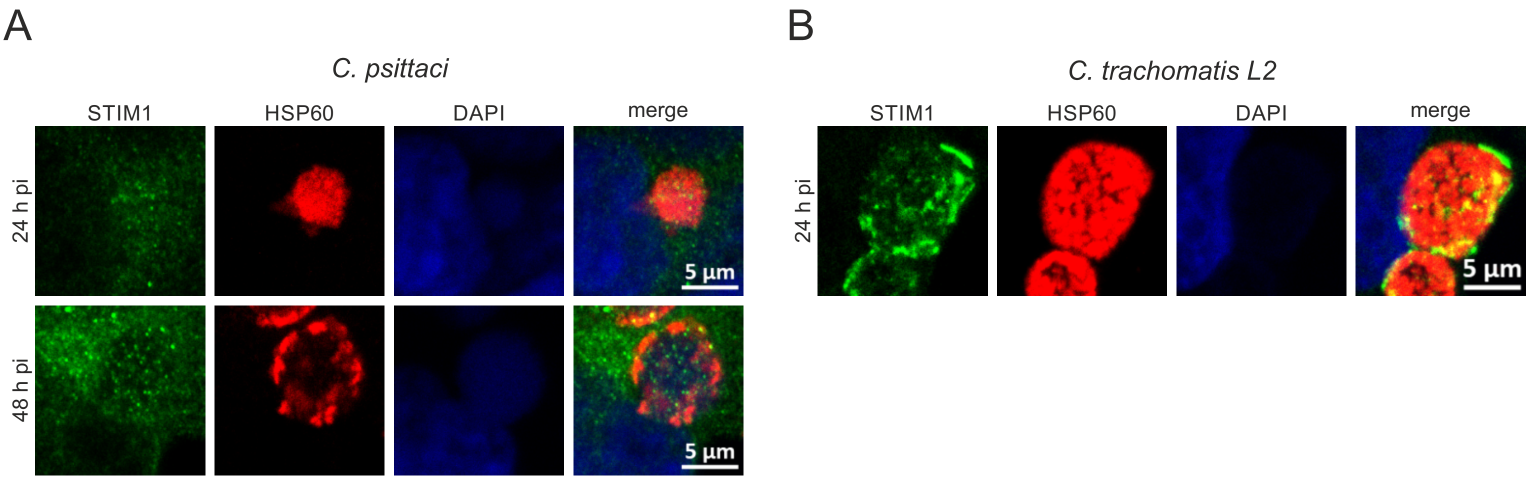

Supplement: Supplementary file 1 — Supplementary Material 1 [file 41598_2025_2077_MOESM1_ESM.pdf]
